# Investigation of fiber utilization in the rumen of dairy cows based on metagenome-assembled genomes and single-cell RNA sequencing

**DOI:** 10.1186/s40168-021-01211-w

**Published:** 2022-01-20

**Authors:** Ming-Yuan Xue, Jia-Jin Wu, Yun-Yi Xie, Sen-Lin Zhu, Yi-Fan Zhong, Jian-Xin Liu, Hui-Zeng Sun

**Affiliations:** 1 Institute of Dairy Science, College of Animal Sciences, Zhejiang University, Hangzhou, 310058 China; 2 Ministry of Education Innovation Team of Development and Function of Animal Digestive System, Zhejiang University, Hangzhou, 310058 China; 3 Ministry of Education Key laboratory of Molecular Animal Nutrition, Zhejiang University, Hangzhou, 310058 China

**Keywords:** Dairy cows, Fiber utilization, Individual microbial genomes, Rumen epithelial cell types, Metagenomic binning, Single-cell RNA sequencing

## Abstract

**Background:**

Dairy cows utilize human-inedible, low-value plant biomass to produce milk, a low-cost product with rich nutrients and high proteins. This process largely relies on rumen microbes that ferment lignocellulose and cellulose to produce volatile fatty acids (VFAs). The VFAs are absorbed and partly metabolized by the stratified squamous rumen epithelium, which is mediated by diverse cell types. Here, we applied a metagenomic binning approach to explore the individual microbes involved in fiber digestion and performed single-cell RNA sequencing on rumen epithelial cells to investigate the cell subtypes contributing to VFA absorption and metabolism.

**Results:**

The 52 mid-lactating dairy cows in our study (parity = 2.62 ± 0.91) had milk yield of 33.10 ± 6.72 kg. We determined the fiber digestion and fermentation capacities of 186 bacterial genomes using metagenomic binning and identified specific bacterial genomes with strong cellulose/xylan/pectin degradation capabilities that were highly associated with the biosynthesis of VFAs. Furthermore, we constructed a rumen epithelial single-cell map consisting of 18 rumen epithelial cell subtypes based on the transcriptome of 20,728 individual epithelial cells. A systematic survey of the expression profiles of genes encoding candidates for VFA transporters revealed that *IGFBP5*^*+*^ cg-like spinous cells uniquely highly expressed *SLC16A1* and *SLC4A9*, suggesting that this cell type may play important roles in VFA absorption. Potential cross-talk between the microbiome and host cells and their roles in modulating the expression of key genes in the key rumen epithelial cell subtypes were also identified.

**Conclusions:**

We discovered the key individual microbial genomes and epithelial cell subtypes involved in fiber digestion, VFA uptake and metabolism, respectively, in the rumen. The integration of these data enables us to link microbial genomes and epithelial single cells to the trophic system.

Video abstract

**Supplementary Information:**

The online version contains supplementary material available at 10.1186/s40168-021-01211-w.

## Background

Meeting the demand for high-quality animal protein has become a global food security concern [1]. Dairy products are optimal animal protein products, and the annual global per capita dairy consumption is over 100 kg [2]. Understanding how dairy cattle convert feed plants into volatile fatty acids (VFAs), and how are the VFAs absorbed by the host is crucial for improving the efficiency of animal production. Such a process largely relies on the symbiotic microbiota in the rumen [3]. Fibers can be broken down by microbes and used for producing VFAs to provide approximately 70% of the cattle’s energy requirements [4]. Studies have reported rumen microbiome-dependent mechanisms explaining the efficiency of converting dietary energy to animal products in dairy cattle [5, 6] and beef cattle [7, 8]. Our previous study demonstrated that rumen microbial taxonomic features and functions affected VFA production, which contributes to host milk performance in dairy cattle [9]. Nevertheless, a system-level understanding of how specific rumen microbe metabolic and carbohydrate-degrading potentials affect fiber processing is still unclear. Although Hungate genomes have increased read classification by 10%, large numbers of rumen microbes are still uncultivated [10]. Metagenomic binning enables the assembly of near-complete microbial genomes directly from metagenomic sequencing data, which significantly improves the microbial reference genomes [10, 11] and read classification (by 50–70%) [12]. However, the fiber digestion and fermentation functions of uncultivated microbial genomes in the rumen of lactating Holstein dairy cattle (one of the most common milk-producing dairy cattle) are still under investigated.

VFAs from the rumen lumen are absorbed and partly metabolized by the stratified squamous rumen epithelium, which is mediated by diverse cell types [13]. The rumen epithelium is composed of living stratum (stratum basale, spinosum, and granulosum) and corneum (dead cornified keratinocytes) [14], and different rumen cell types execute distinct functions. Studies using cell cultures showed that basale and spinosum strata cells play essential roles in VFA uptake and metabolism [15, 16]. Previous research based on bulk RNA sequencing of rumen tissues discovered that specific VFA metabolic genes and processes in the rumen epithelium were enriched [17]. Investigations on rumen epithelial cell subtypes and specific roles in VFA absorption and metabolism using traditional molecular methods is still a major challenge. Although direct observations of cell functions *in vivo* are difficult at the single-cell level, it is commonly accepted that single-cell RNA sequencing (scRNA-seq), an approach for exploring the gene expression features of cells [18, 19], could provide new insights into cell type-specific functions at single-cell resolution.

In the current study, we applied metagenomic binning to explore the individual microbes involved in fiber digestion and fermentation, and further performed scRNA-seq on rumen epithelial cells to investigate the accurate cell subtypes contributing to VFA uptake and metabolism. The integration of these data enables us to link microbial genomes and epithelial single cells to the trophic system. Our current study provides a novel understanding of the specialization of fiber utilization in the rumen system at individual microbial taxa and epithelial single-cell resolution.

## Results and discussion

### Bacterial taxonomic composition of metagenome-assembled genomes (MAGs)

Metagenomic sequencing generated 2,751,185,494 reads from a total of 49 lactating dairy cows (Supplementary Table S1). After low-quality reads and host genes were removed, a total of 2,697,595,628 clean reads were generated, 1,002,914,596 of which were annotated to the RefSeq database, with 941,151,278 reads annotated to bacteria. The metagenomic sequencing data (415 GB) were further assembled into 34,039,290 contigs and used to reconstruct MAGs. After filtering MAGs based on completeness and contamination, a total of 186 rumen microbial MAGs were obtained from our dataset, with completeness > 70% (88.22% ± 9.45%) and contamination < 10% (5.81 ± 2.71%) (Supplementary Table S2). These MAGs represent 80.14% of the total number of reads in the metagenomes. All 186 MAGs had < 95% average nucleotide identity (ANI), which means that these 186 species were nonredundant. Among them, 92 MAGs were near complete (completeness ≥ 90%), and 94 MAGs were substantially complete (70% ≤ completeness ≤ 90%) according to the definition of high-quality MAGs proposed by Parks et al. [20]. A total of 71 out of these 186 MAGs displayed lower contamination (contamination < 5%) according to the criteria of Parks et al. [20]. The genome sizes of these 186 MAGs ranged from 659,879 bp to 26,548,065 bp (5,867,423 ± 5,008,174 bp, mean ± SD), with N50 values ranging from 2108 bp to 61,696 bp. The average number of tRNA genes of each MAG was 21.36 ± 11.39% (ranging from 1 to 64), which transferred 11.84 ± 4.43% amino acids (ranging from 1 to 20).

**Fig. 1:**
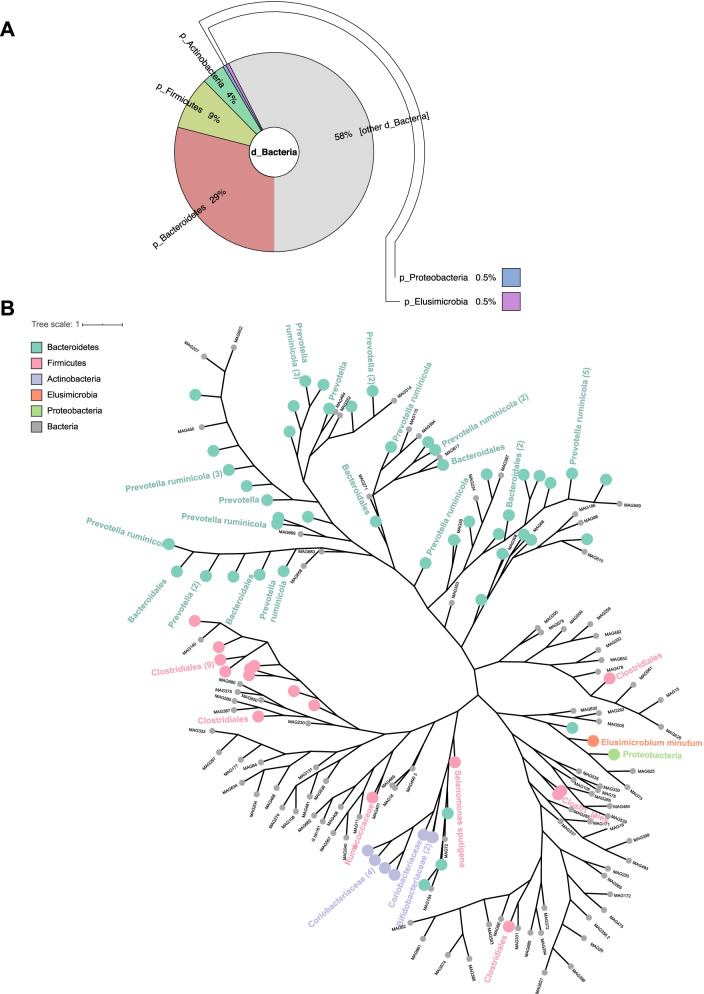
Profiles of 186 rumen MAGs recovered from 49 dairy cows based on metagenomic binning analysis. **A** Taxonomic composition of 186 rumen MAGs at the phylum level. **B** Phylogenetic tree of 186 MAGs from the dairy cattle rumen. Color nodes: MAGs classified to different taxonomic levels; gray nodes: MAGs classified to the kingdom level. MAGs: metagenome-assembled genomes

The MAGs were then taxonomically assigned, with all 186 MAGs assigned to bacteria (Fig. 1A), and the majority of these MAGs belonged to uncultivated linages without any previous metabolic or phylogenetic characterization. Overall, 79 MAGs could be taxonomically assigned to five phyla: *Bacteroidetes* (54 MAGs), *Firmicutes* (16 MAGs), *Actinobacteria* (7 MAGs), *Proteobacteria* (1 MAG), and *Elusimicrobia* (1 MAG). The remaining 57.53% of bacterial MAGs belonging to phyla lacked existing sequenced representatives (Fig. 1A). At the order level, bacterial MAGs were assigned into six orders, including *Bacteroidales* (40 MAGs), *Clostridiales* (14 MAGs), *Coriobacteriales* (5 MAGs), *Bifidobacteriales* (2 MAGs), *Elusimicrobia* (1 MAG), and *Selenomonadales* (1 MAG) (Fig. 1B and Supplementary Figure S1). At the family level, MAGs were assigned to six bacterial families: *Prevotellaceae* (27 MAGs), *Coriobacteriaceae* (5 MAGs), *Bifidobacteriaceae* (2 MAGs), *Elusimicrobiaceae* (1 MAG), *Ruminococcaceae* (1 MAG), and *Veillonellaceae* (1 MAG) (Fig. 1B and Supplementary Figure S1). We resolved three of the MAGs to species, including *Prevotella ruminicola*, S*elenomonas sputigena*, and *Elusimicrobium minutum*.

As our study and previous studies have shown, metagenomic binning is an effective technique to recover rumen microbial genomes (complete or near-complete) without culture procedures [3, 10, 12]. Given that culture-based methods could not provide a complete understanding of rumen microbes due to the strictly anaerobic environment of the rumen, this novel tool enables researchers to understand the function of any microbiome and link rumen microbes to phenotypes easily and effectively. To fully understand the functions of rumen microbes for future improvement interventions of production traits, it is vital to recover the microbes to the species and even strain level. However, consistent with the results of previous metagenomic binning studies, our results showed that a large number of unclassified microbial genomes and genomes could not be resolved to the species level, with many MAGs recovered at only the kingdom level [3, 10, 12]. Previous studies reporting the phylogenetic diversity census of rumen microbiomes have also highlighted that unclassified bacteria are the most abundant rumen microorganisms [11, 21, 22]. These results suggest that there are still a large number of microbial genomes yet to be sequenced and assembled.

### CAZome profiles of MAGs

**Fig. 2:**
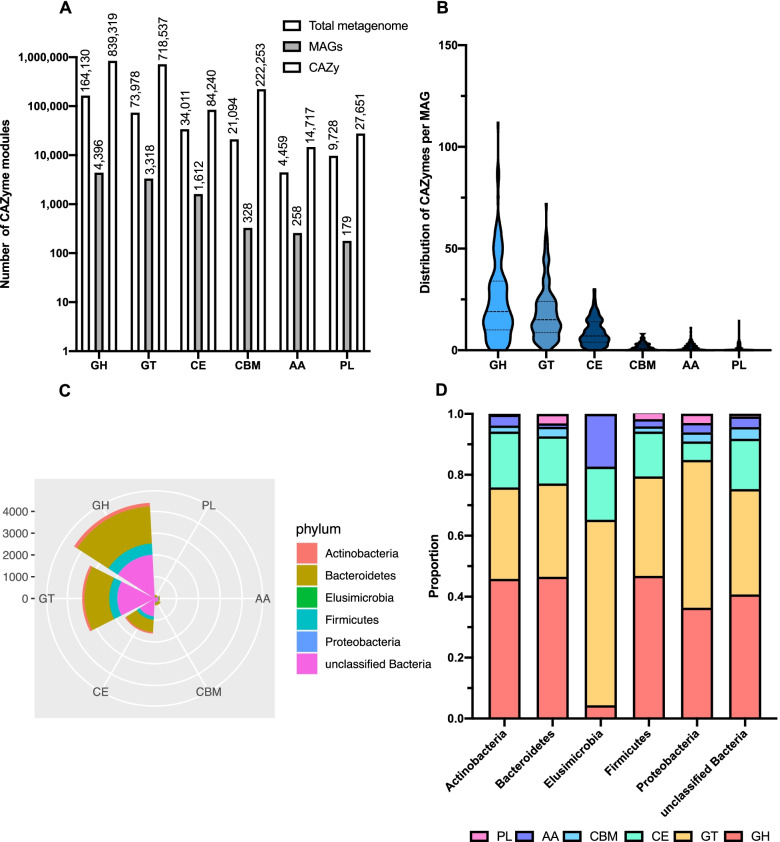
CAZyme profiles of the rumen MAGs. **A** The number of each CAZyme module predicted in the total metagenome datasets and the MAGs and their representation in the CAZy database at the time of submission. **B** The distribution numbers of six CAZyme modules encoded in each MAG. **C** Phylum-level taxonomic distribution of predicted CAZyme modules. **D** Distribution of six CAZyme classes as a proportion of the total number of predicted CAZymes in each phylum. MAGs: metagenome-assembled genomes.

Breakdown of complex plant fibers is initiated by degradative enzymes of rumen microbes, including enzymes capable of degrading cellulose, xylan (hemicellulose), and pectin. We predicted CAZyme modules based on both metagenomic datasets and MAGs to characterize the profile of carbohydrate-active enzymes present in dairy cow rumen. The most frequently occurring GH modules included GH2 (19,219), GH13 (15,765), GH43 (11,093), GH3 (6871), GH97 (5760), GH31 (5314), and GH5 (5,308) (Supplementary Table S3). A total of 2,964 GH families involved in cellulose degradation were identified, including GH5, GH9, GH44, GH45, and GH48. A total of 13,110 pectin-degrading families were identified, including GH28, PL1, PL9, PL10, PL11, CE8, and CE12. The number of CAZymes involved in xylan degradation was 24,488, and these CAZymes included GH8, GH10, GH11, GH43, GH51, GH67, GH115, GH120, GH127, CE1, and CE2. We then compared CAZymes predicted in the total metagenome with those in the public database, with Fig. 2A showing the numbers of each CAZyme class predicted in the total metagenome as well as the representation of each CAZyme class in the CAZy database (31 July 2020).

In total, the MAGs contained 43968 GHs, 3318 GTs, 1612 CEs, 328 CBMs, 258 AAs, and 179 PLs (Fig. 2A). Figure 2B shows the count distribution of the six CAZyme classes in MAGs. On average, each MAG contained 24.15 ± 19.59 GHs (ranging from 0 to 112), 18.23 ± 13.22 GTs (ranging from 0 to 72), 8.86 ± 6.44 CEs (ranging from 0 to 30), 1.80 ± 1.89 CBMs (ranging from 0 to 8), 1.42 ± 1.77 AAs (ranging from 0 to 11), and 0.98 ± 2.21 PLs (ranging from 0 to 15). The taxonomic and functional distribution of proteins in MAGs (Fig. 2C) revealed that unknown bacteria contained the highest number of CAZymes, followed by *Bacteroidetes* and *Firmicutes*. The distribution of CAZymes across bacterial phyla (Fig. 2D) showed that GHs, GTs, and CEs contributed the largest number of CAZymes of most phyla and unknown bacteria (not including *Elusimicrobia*). Characterization of the CAZyme profiles highlighted the carbohydrate degradation and utilization strategies used by different microbial taxa detected in our collection.

### Fiber-degrading capabilities of MAGs belonging to *Bacteroidetes*

**Fig. 3:**
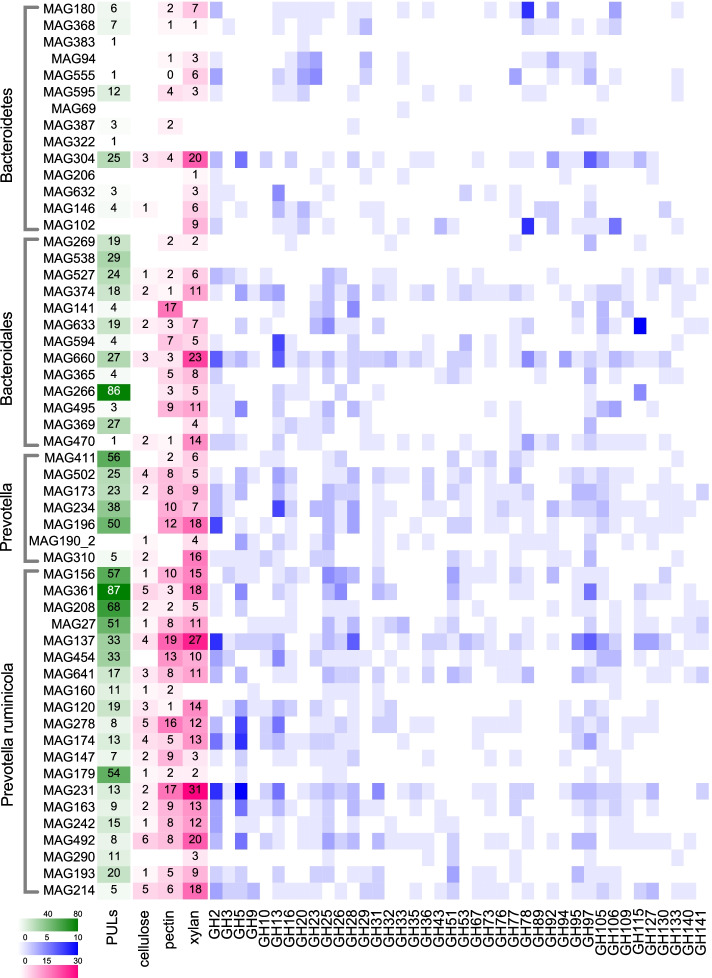
The polysaccharide degradation potential of MAGs belonging to *Bacteroidetes*. A heatmap showing counts of polysaccharide-degrading CAZymes and selected GH modules encoded in the MAGs, as well as counts of polysaccharide utilizing loci (PULs) in the MAGs. Cellulose degradation: GH5, GH9, GH44, GH45, GH48; pectin degradation: GH28, PL1, PL9, PL10, PL11, CE8, CE12; xylan degradation: GH8, GH10, GH11, GH43, GH51, GH67, GH115, GH120, GH127, CE1, CE2. MAGs: metagenome-assembled genomes

Our MAG data revealed the vital saccharolytic role of *Bacteroidetes* in rumen carbohydrate degradation, as the MAGs of this dominant phylum encode proteins capable of binding and digesting multiple carbohydrate substrates. The *Bacteroidetes* MAGs encoded 38.85% of the total GHs (the group of enzymes that hydrolyze glucoside bonds between carbohydrates) detected in our study. We then profiled the distribution of GH modules as well as important fiber-degrading capabilities of cellulose degradation, pectin degradation, and xylan degradation across 54 *Bacteroidetes* MAGs (Fig. 3). The top 10 most frequently occurring GH families in *Bacteroidetes* genomes were GH2, GH5, GH13, GH97, GH25, GH28, GH78, GH105, GH106, and GH23. In addition, important cellulose-degrading CAZymes, pectin-degrading CAZymes, and xylan-degrading CAZymes were mostly encoded by *Prevotella* at the genus level (including MAG234 and MAG196) and *P. ruminicola* at the species level (including MAG156, MAG361, MAG137, MAG278, MAG174, MAG231, MAG492, and MAG214) (Fig. 3).

CAZymes are often located within polysaccharide utilization loci (PULs), gene clusters that encode enzymes that are necessary for bacteria to bind, transport, and depolymerize specific glycan structures [23]. Taxa of the phylum *Bacteroidetes* evolve PULs, with GH modules most frequently organized in PULs [24, 25]. As PULs are specific to one particular or multiple substrates, the observed PULs in MAGs can be applied to predict substrates of microbial genomes [26]. We then detected the PULs in *Bacteroidetes* MAGs, and the detailed information is presented in Supplementary Table S4 and Supplementary Figure S2. A total of 1827 putative PULs were identified from all *Bacteroidetes* MAGs. Among these *Bacteroidetes* MAGs, 50 contained more than one PUL, and the number of PULs per MAG varied from 1 to 87. The PUL profile revealed that the most common CAZyme-associated PULs were those involved in xylan degradation (GH43), pectin degradation (GH28), starch degradation (GH13 and GH97), and accessory enzymes involved in plant polysaccharide digestion and the degradation of carbohydrates originated from microbes in the rumen (GH2, GH15, GH32).

Several MAGs that contained the most abundant PULs clustered together with *P. ruminicola* (MAG156, MAG361, MAG208, MAG27, and MAG179, 99% ANI), an anaerobic gram-negative bacterium belonging to *Bacteroidetes* that utilizes multiple polysaccharides as substrates [27, 28] (Fig. 3). This branch of the phylogenetic tree might be microbes that contain novel polysaccharide-degradation active enzymes. In our study (Supplementary Table S4), the CAZymes present in PULs detected in *P. ruminicola* were mainly alpha-glucosidase (EC:3.2.1.20), arabinan endo-1,5-alpha-L-arabinosidase (EC:3.2.1.99), beta-fructofuranosidase (EC:3.2.1.26), beta-galactosidase (EC:3.2.1.23), endoglucanase (EC:3.2.1.4), fructan beta-fructosidase (EC:3.2.1.80), mannan endo-1,4-beta-mannosidase (EC:3.2.1.78), para-nitrobenzyl esterase (EC:3.1.1.-), and pectinesterase (EC:3.1.1.11), indicating that these MAGs potentially have strong saccharolytic capability and may be able to adapt to the rumen under multiple diets. Stewart et al. reported that *P. ruminicola* was the second largest contributor of PULs (containing fewer PULs than *P. multisaccharivorax*), with enzymes encoded in PULs of *P. ruminicola* in that study including pectate lyases, pectin esterases, glucosidases, arabinases, galactosidases, and mannosidases [12]. The inconsistency of the largest source of PULs in our study (*P. ruminicola*) and that of Stewart et al. (*P. multisaccharivorax*) may be due to the different breeds and diets, as breed and diet are two critical impact factors affecting rumen microbiome composition [8, 29, 30]. The MAGs in Stewart et al. were recovered from cows from three cross breeds (including Aberdeen Angus, Limousin and Charolais) and one pure breed (Luing) that were fed two diets, including a high-concentrate diet (forage: concentrate = 75: 925, dry matter-based) and mixed forage-concentrate diet (forage: concentrate = 480: 520) [31], whereas the MAGs in our study were generated from Holstein dairy cows that were fed a corn-based high-grain diet (forage:concentrate = 450:550). In the current study, *P. ruminicola* having the most PULs suggests the high polysaccharide degradation potential of this clade [12, 31], which allows the use of multiple substrates in the rumen of Holstein dairy cows and may act as a vital intermediate metabolite contributor to milk biosynthesis.

### Epithelial single-cell map of the rumen in lactating dairy cows

**Fig. 4:**
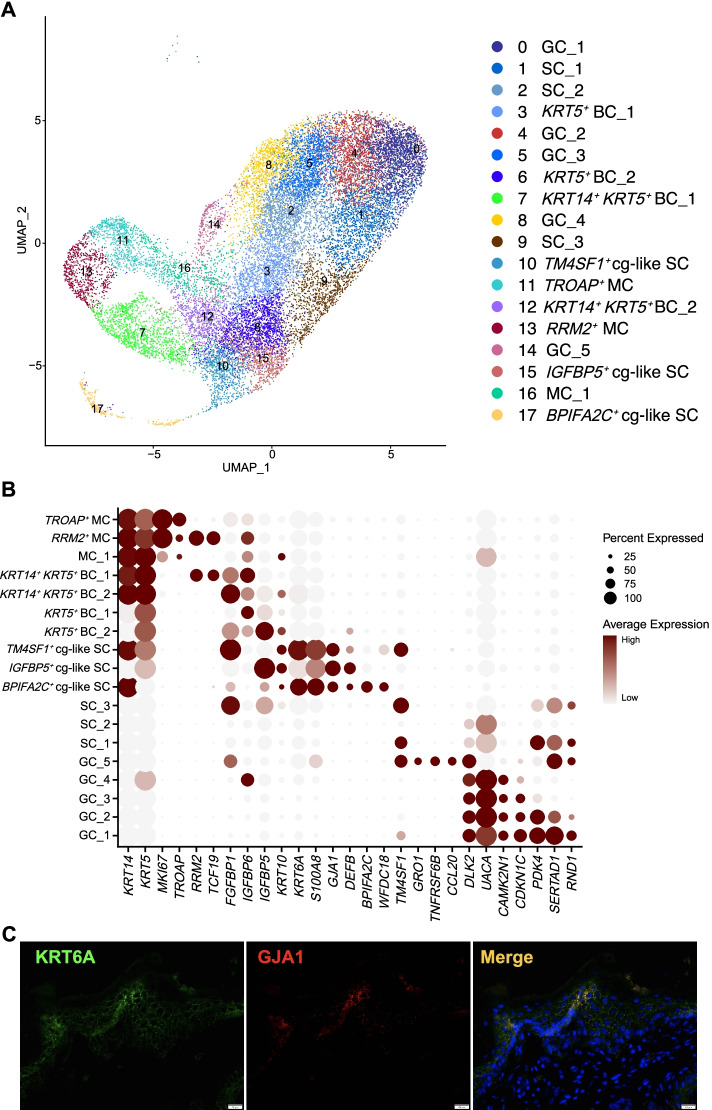
Epithelial single-cell map of the rumen in lactating Holstein dairy cows. **A** UMAP map of rumen epithelial single cells showing the 18 cell types of rumen epithelial cells. Cells are colored by cell type. **B** Dot plot visualization of each cell type in the rumen epithelial single-cell map. Dot size represents the percentage of cells within a cell type, and the color refers to the expression level. **C** Coimmunostaining of KRT6A with GJA1 in rumen tissues. Scale bars, 20 μm. Experiments were independently repeated twice for each tissue, with similar results. UMAP: Uniform Manifold Approximation and Projection; BC: basal cell; SC: spinous cell; GC: granulear cell

As the limited availability of useful flow cytometry reagents with specificity to rumen epithelial cell types has hindered sorting cells for in vitro functional assays and directly investigating the functions of rumen epithelial cell types in vivo is difficult, the type of epithelial cells responsible for the uptake of VFAs in the rumen epithelium is still largely unknown. Recent advances in scRNA-seq enable the expression profiling of individual cells and are also an indirect means of assessing cell type-specific functions. Herein, we performed scRNA-seq analysis of 20,728 high-quality individual epithelial cells from ventral rumen tissues of 3 lactating Holstein dairy cows (Fig. 4A). Our results showed that cells from the 3 cows overlapped well (analysis of similarities statistic *R* = − 0.012 and *P* = 1) (Figures S4A–B), indicating the high fidelity of the data and reproducibility of the rumen epithelial cellular landscapes obtained from the three individuals. In total, we discovered 18 rumen epithelial cell clusters in lactating dairy cows (Fig. 4A). Clusters 11, 13, and 16 were proliferating basal cells (mitotic cell, MC) expressing the marker genes *KRT14*, *KRT5*, and *MKI67* [32], and were divided into three subpopulations of MC (*TROAP*^*+*^ MC, *RRM2*^*+*^ MC, and MC_1, respectively) based on their highly expressed specific genes (Fig. 4B and Figure S5A). Clusters 3, 6, 7, and 12 highly expressed the basal cell (BC) markers *KRT14* or *KRT5* [33] and were defined as four subtypes of BC (*KRT5*^*+*^ BC_1, *KRT5*^*+*^ BC_2, *KRT14*^*+*^ *KRT5*^*+*^ BC_1, and *KRT14*^*+*^ *KRT5*^*+*^ BC_2, respectively) characterized by their highly expressed specific genes (Fig. 4B and Figure S5B). Clusters 0, 4, 5, 8, and 14 were granule cell (GC) types with high levels of *DLK2* (Fig. 4B), whose expression is restricted to the granule layer and acts as a key regulatory factor of keratinocyte terminal differentiation and cornification [34]. By comparison, we identified the highly expressed genes in these five GC subtypes (Figure S5C). Clusters 1, 2, and 9 were predicted to be three spinous cell subtypes (SC_1-3) because they did not express the basal cell markers (such as *KRT14* and *KRT5*) but slightly expressed the spinous cell marker *KRT10* [33] or granule cell marker *DLK2* (Fig. 4B). These indicated that they may serve as the spinous cell types between basal cell and terminally differentiated granulear cell, and we also identified their highly expressed genes (Figure S5D). Interestingly, in addition to high levels of the spinous cell marker genes *KRT10*, *S100A8*, and *KRT6A*, clusters 10, 15, and 17 specifically expressed *GJA1* (a channel-gap cell gene marker) (Fig. 4B and Supplementary Table S5) [33]; therefore, they were defined as cg-like SCs (channel-gap like spinous cells), which was further verified by immunofluorescence staining (Fig. 4C). The cg-like SCs were further classified into different subtypes such as *TM4SF1*^*+*^ cg-like SC (cluster 10), *IGFBP5*^*+*^ cg-like SC (cluster 15), and *BPIFA2C*^*+*^ cg-like SC (cluster 17) with *TM4SF1* (*P*_adj = 8.26e−90), *IGFBP5* (*P*_adj = 2.92e-251), and *BPIFA2C* (*P*_adj = 4.04e-139) based on their respective highly expressed genes (Fig. 4B and Supplementary Table S5). We also revealed that these three cg-like SCs all highly expressed *GSTA1* and *TMEM79* (Figure S5E and Supplementary Table S5). *GSTA1* encodes an enzyme that protects cells from reactive oxygen species [35], and *TMEM79* plays a vital role in epidermal integrity and barrier function [36]. The high level of *FABP4* (Figure S5E and Supplementary Table S5), a fatty acid binding protein gene [37], in these three cg-like SCs indicates that they are involved in fatty acid absorption, transport, and metabolism.

### Key cell subtypes responsible for VFA absorption

**Fig. 5:**
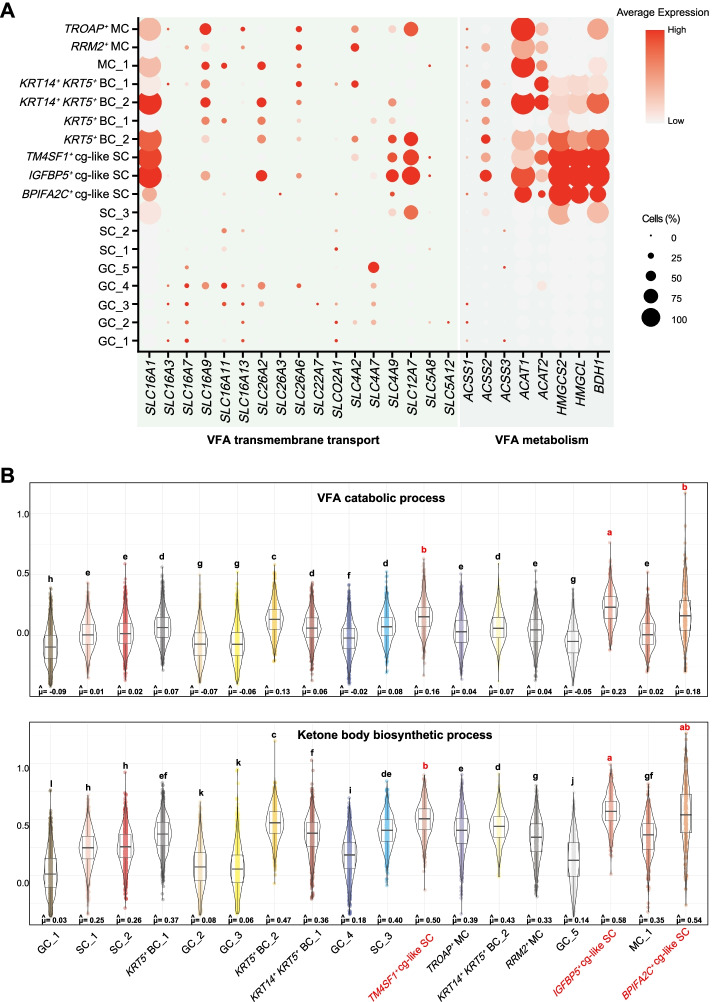
Functional heterogeneity of VFA absorption and metabolism across rumen epithelial cell subtypes. **A** Dot plot showing the expression of genes that might encode transporters of VFA absorption and metabolism for all rumen epithelial cell subtypes. The node size represents the percentage of cells within a cell type. The color transition from gray to red indicates low to high gene expression levels. **B** Gene scoring analysis of rumen cell subtypes using the VFA catabolic process and ketone body biosynthetic process gene sets. VFAs: volatile fatty acids

Anion exchange mechanisms play vital roles in the uptake of VFAs from the lumen into the epithelium and extrusion into the blood. There are numerous molecular candidates for anion exchange-dependent and anion exchange-independent VFA transport, including those of the SLC16A, SLC26A, SLC22A, SLC21A, SLC4A, and SLC5A families [17, 38–40]. We analyzed the expression of genes within the above families in each cell type and found that the expression pattern of these genes was cell type specific, although some of these genes (such as *SLC16A3*, *SLC16A7*, *SLC16A9*, *SLC16A11*, *SLC16A13*, *SLC26A3*, *SLC26A6*, *SLC22A7*, *SLC02A1*, *SLC5A8*, and *SLC5A12*) had relatively low percentages of expression (Fig. 5A). *SLC16A9* was mainly present in the BC subtypes, and *SLC26A2* was detected in MC_1, *KRT14*^*+*^ *KRT5*^*+*^ BC_2, and *IGFBP5*^*+*^ cg-like SC, although no significant difference was found. *SLC4A7* was uniquely highly expressed in GC_5 (*P*_adj = 4.46e−70). *KRT14*^*+*^ *KRT5*^*+*^ BC_2 (*P*_adj = 5.79e−150), *KRT5*^*+*^ BC_2 (*P*_adj = 1.71e−248), *TM4SF1*^*+*^ cg-like SC (*P*_adj = 9.67e-169), and *IGFBP5*^*+*^ cg-like SC (*P*_adj = 2.23e-281) highly expressed *SLC16A1* (Fig. 5A and Supplementary Table S5), which has a vital role in VFA absorption [41]. Moreover, *SLC4A9*, encoding the most likely VFA transporter [17], was detected with a high expression level in the *IGFBP5*^*+*^ cg-like SC (*P*_adj = 2.78e−77) (Fig. 5A). *SLC4A9* was also present in *KRT5*^*+*^ BC_2 and *TM4SF1*^*+*^ cg-like SC, although no significant difference was found. These results suggest that VFAs may rarely be taken up by the cell subtypes of GC, even though these cells are the living cells that anatomically first contact VFAs produced in the ruminal lumen. *KRT5*^*+*^ BC_2, *TM4SF1*^*+*^ cg-like SC, and *IGFBP5*^*+*^ cg-like SC highly expressed the genes encoding the candidates for VFA transporters and played more important roles in VFA uptake. The mechanism by which VFAs pass through granule cells to reach cells in the stratum basale and spinosum needs to be further elucidated.

### VFA metabolism heterogeneity across rumen epithelial cell subtypes

VFA metabolism is another important function of VFA utilization executed by rumen epithelial cells. Next, we investigated the patterns of genes related to VFA metabolism (*ACSS1*, *ACSS2*, *ACSS3*, *ACAT1*, *ACAT2*, *HMGCS2*, *HMGCL*, and *BDH1*) in rumen epithelial cell types [39, 42]. *ACSS1*, *ACSS2*, and *ACSS3* were reported to activate VFA metabolism [39]; however, only *ACSS2* was detected in *KRT5*^*+*^ BC_2 and *IGFBP5*^*+*^ cg-like SC (Fig. 5A). *ACAT1* was expressed at a high percentage and at high levels in *TROAP*^+^ MC (*P*_adj = 1.79e−177), MC_1 (*P*_adj = 7.73e−126), *KRT14*^*+*^ *KRT5*^*+*^ BC_2 (*P*_adj = 2.69e−108), and *IGFBP5*^*+*^ cg-like SC (*P*_adj = 1.80e−90) (Fig. 5A and Supplementary Table S5). *HMGCS2*, *HMGCL*, and *BDH*, which are involved in the synthesis of ketone bodies [42], were highly expressed in *KRT5*^*+*^ BC_2 and the three cg-like SC subtypes (Fig. 5A and Supplementary Table S5). Collectively, these results showed that *TROAP*^+^ MC, MC_1, *KRT14*^*+*^ *KRT5*^*+*^ BC_2, *KRT5*^*+*^ BC_2, *TM4SF1*^*+*^ cg-like SC, *BPIFA2C*^*+*^ cg-like SC, and especially *IGFBP5*^*+*^ cg-like SC are the major cell subtypes response for VFA metabolism. This was further confirmed by the gene set scoring analysis, which showed that the *IGFBP5*^*+*^ cg-like SC had the largest score for both the “VFA catabolic process” (μ = 0.23) and “ketone body biosynthetic process” (μ = 0.58) (Fig. 5B).

### MAGs and epithelial cells involved in rumen fiber utilization

In the rumen system, fiber utilization is performed through interconnection of microbial metabolism and epithelial cell functions. To understand the carbohydrate degradation activities and metabolic potential of cow rumen microbes as well as the potential of VFA absorption and metabolism of rumen epithelial cells, we integrated our results.

**Fig. 6:**
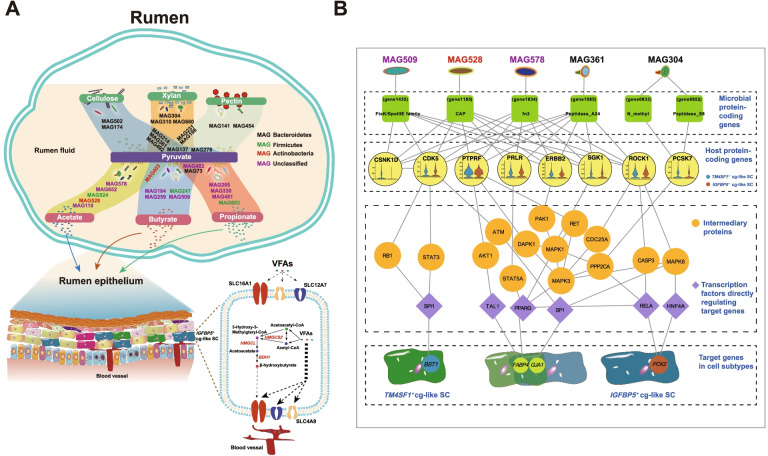
Proposed model showing the metabolic potentials of bacterial genomes and their interactions with rumen epithelial cells. **A** Different colors of MAGs represent microbes belonging to different phyla. The schematic on the lower left represents the composition of the cells in the rumen epithelium, and the uptake and metabolism of VFAs in *IGFBP5*^+^ cg-like SC is shown on the lower right. **B** Network obtained by retaining only signaling pathways with transcriptional regulatory interactions between the intermediary protein and the target genes

Figure 6A shows the potential for the degradation of plant structural carbohydrates and VFAs by the key bacterial MAGs. Based on linkages to specific substrate classes, MAGs were assigned to at least one fiber deconstruction and fermentation pathway. Genomes that we solely detected strongly encoded proteins for the deconstruction of recalcitrant polymers (cellulose, xylan, and pectin) included MAG502 and MAG174 (cellulose); MAG304, MAG310 and MAG660 (xylan); and MAG141 and MAG454 (pectin). Notably, several genomes played important roles in encoding more than one substrate type. For example, MAG137 (a *Prevotella* sp. genome) had strongly encoded proteins for the degradation of all three types of substrates, which could be further investigated for sourcing carbohydrate-degrading enzymes from the rumen for use as animal feed additives and lignocellulose-based biofuel generation. For the utilization of intermediate metabolites (pyruvate) to produce VFAs, several genomes showed strong capabilities, with MAG403 (involved in acetate and butyrate biosynthesis) and MAG482 and MAG73 (involved in butyrate and propionate biosynthesis) functioning in more than one VFA metabolism pathway. Based on this functional scheme, we obtained a better understanding of which microbial group encoded these vital functions essential for carbohydrate utilization and metabolism in the rumen. Additionally, this model provides us with a fundamental framework for understanding gene functions in these microbial genomes and for designing future strategies for manipulating rumen microbes for improving VFA production and other traits. Because these metagenomic binning-based functions are potential rather than real functions, future studies investigating the causations behind the potential functions of MAGs (e.g., culture-based investigations) are required.

On the other hand, efficient mechanisms for the transport of VFAs were crucial for the efficiency of animal production. Over 90% of VFAs are present as VFA anions that are absorbed by epithelial cells from the lumen to blood in different manners, especially through protein-mediated transport mechanisms [43]. Unlike *SLC16A1* and *SLC4A9*, *SLC12A7*, which is responsible for chloride transport [44], was also highly expressed in the *IGFBP5*^*+*^ cg-like SC (Fig. 5A and Supplementary Table S5, *P*_adj = 3.00e−72). Previous studies found that the uptake of VFAs, especially propionate, was negatively correlated with the disappearance of chloride; thus, the involvement of outwardly rectified Cl^−^ channels was also proposed [38, 43]. Intracellular breakdown of VFAs, especially butyrate, in the *IGFBP5*^*+*^ cg-like SC could maintain the concentration gradient for VFAs across the rumen epithelium to ensure luminal uptake [45]. Taken together, these results suggest that this epithelial cell type may play an important role in the uptake of VFAs.

### Inferring functional effects of microbiome-host interactions

In addition to the metabolite-mediated interactions between microbes and host cells, protein-protein interactions are one of the most relevant types of molecular interplay that can modulate the expression of genes of host cells [46, 47]. However, there is a dearth of information on protein-protein interactions between the microbiome and ruminal epithelial cells in publicly available databases because experimental techniques for testing interspecies protein–protein interactions are time-consuming and costly. Here, we used MicrobioLink [48] to identify potential cross-talk between microbial and bovine proteins and how these interactions modulate the expression of key genes in rumen epithelial cell subtypes. First, 223 secreted microbial proteins (Supplementary Table S6) were obtained from the 30 key MAG sequences mentioned above. The proteins located in the cellular membrane and extracellular matrix were identified using UniProt [49], resulting in a total of 8169 proteins (Supplementary Table S7). A total of 2949 interactions (Supplementary Table S8) involving 9 microbial proteins and 750 host receptor proteins were predicted using the “domain-domain interaction prediction” function implemented in MicrobioLink. As host potential target genes were affected by the microbiome-host interactions, we focused on the top 5 genes involved in fatty acid metabolism that were significantly highly expressed in the *IGFBP5*^*+*^ cg-like SC and *TM4SF1*^*+*^ cg-like SC (*GJA1*, *GSTA1*, *FABP4*, *FABP5*, and *ACSF2*; avg_logFC = 1.84 and 1.35; 1.58 and 2.87; 1.25 and 1.42; 1.17 and 1.73; and 0.76 and 0.64, respectively; all *P*_adj <0.05) and the top 5 genes that were uniquely highly expressed in these two cell subtypes (*CCDC80*, *AK1*, *CA12*, *PCK2*, and *FPGS* in the *IGFBP5*^*+*^ cg-like SC; and *AGPAT2*, *ZNF750*, *CD82*, *BST1*, and *ATP6V1B1* in the *TM4SF1*^*+*^ cg-like SC) compared to other cell subtypes. Subsequently, the potential signaling networks mediating signal transduction from the host receptors to the target genes were constructed. To retain specificity, the signaling pathways initiated from host proteins that were not detected in either *IGFBP5*^*+*^ cg-like SC or *TM4SF1*^*+*^ cg-like SC were excluded. Finally, we obtained a network with 4 of the 15 target genes that could be potentially modulated by the microbiome (Fig. 6B). We observed a signaling pathways specific to regulating the expression of *BST1* in *TM4SF1*^*+*^ cg-like SC that included CSNK1D, CDK5, STAT3, RB1, and SPIL. The pathway was modulated by the microbial protein labeled gene1435 and uniquely found in MAG509. Expression of the gene encoding CSNK1D was detected in the *TM4SF1*^*+*^ cg-like SC but not in the *IGFBP5*^*+*^ cg-like SC (Fig. 6B). MAG361, MAG528, MAG578, MAG509, and MAG304 were involved in modulating the expression of *GJA1* and *FABP4* in the *TM4SF1*^*+*^ cg-like SC and *IGFBP5*^*+*^ cg-like SC in the signaling networks (Fig. 6B). The potential protein-protein interactions between the microbes and ruminal epithelial cells identified in our study were based on MAGs and RNA sequencing data (epithelial cells). Future studies are required to examine the expression of the proteins of microbes and host cells to further validate such cross-talk between microbial and bovine proteins, and explain how these interactions modulate the expression of key genes in rumen epithelial cell subtypes. Since protein-protein interactions are one of the most relevant types of molecular interplay that can modulate the expression of genes of host cells [46, 47], our integrated analysis helps to infer indirect effects of microbial proteins on host cells by a signaling network in addition to the metabolite-mediated interactions between microbes and rumen epithelial cells. All the data in the present study are publicly available (for details, see “Availability of data and materials” section) to provide a comparative framework for future studies using the model of ruminant animals.

## Conclusions

The recovery of rumen microbial genomes from Holstein dairy cows provides novel insights into microbial genomes that lack cultivated representatives at the genus or family levels. This knowledge provides a foundation for modeling and developing an understanding of fiber deconstruction by rumen microbes for sourcing carbohydrate-degrading enzymes from the rumen. Furthermore, our rumen epithelial single-cell analysis revealed cell type heterogeneities in VFA absorption and metabolism in which *IGFBP5*^*+*^ cg-like SCs were specifically involved in these functions. Our data provide fundamental knowledge of lactating dairy cows, which enables future interventions for selecting and manipulating the metabolism of microbes and the absorption of VFAs for better performance and health of ruminant animals. Nevertheless, functional assays targeting new findings should be considered in the future.

## Methods

### Sampling and metagenomic sequencing

All experimental procedures of the current study were approved by the Animal Care Committee of Zhejiang University (approval number ZJU20170422) and were performed under the university’s guidelines for animal research.

A total of 52 Holstein dairy cows (parity = 2.62 ± 0.91, days in milk = 167.50 ± 29.98) were selected from commercial dairy farms in the same area in Hangzhou, China. All the animals were fed a corn-based high-grain diet, with a forage-to-concentrate ratio of 45:55, which represents the most common feeding strategy in this area. As previously described, rumen content samples of each animal were collected using oral stomach tubes following a previous protocol [50] before morning feeding [51, 52]. Total microbial DNA was extracted from rumen contents following the protocol of the repeated bead-beating plus column method [53]. Metagenomic libraries were constructed using TrueSeq DNA PCR-Free Library Prep Kits (Illumina, San Diego, USA) and sequenced on an Illumina HiSeq 3000 platform (150 bp paired-end) by Majorbio Biopharm Technology Co., Ltd. In total, 49 samples were sequenced.

### Assembly and functional annotation from rumen metagenomes

Metagenomic data processing and assembly were performed as previously described [9]. In brief, after quality filtering using Fastp (v0.20.0, http://opengene.org/fastp/), each sample was de novo assembled separately using MEGAHIT (v1.2.9, https://github.com/voutcn/megahit) with a minimum contig size of 500 bp [54]. Open reading frames were predicted from these assembled contigs using MetaGeneMark (v3.25, http://exon.gatech.edu/meta_gmhmmp.cgi) [55]. Assembled contigs were pooled to construct nonredundant sequences based on the identical contigs using CD-HIT (v4.8.1, https://github.com/weizhongli/cdhit) [56]. Original sequences were mapped to predicted genes to estimate abundances using SOAPaligner (v2.21, http://soap.genomics.org.cn/) [57]. Contigs were annotated against the CAZy database (dbCAN2 version, 31 July 2020) using USEARCH (v10, http://www.drive5.com/usearch/) [58] and against the KEGG database (Release 95, 1 July 2020) using DIAMOND (v0.9.35, https://github.com/bbuchfink/diamond), with a cutoff of e value < 1 × 10^−5^ [59].

### Metagenomic binning

Metagenomic binning was performed using MetaBAT2 (v2.12.1, http://bitbucket.org/berkeleylab/metabat) [60] on each sample individually. Contigs longer than 3 kb from these 49 metagenomes were used for binning, setting the minimum contig depth at 2 and minimum contig length at 1000. Coverage values across the 49-metagenome dataset were taken into account. The “Outliers” method of RefineM (v0.1.1, https://github.com/dparks1134/RefineM) was used for filtering MAGs by evaluating the GC content, coverage and tetranucleotide frequency of contigs in each MAG, correcting the MAGs by deleting contaminated sequences. dRep (v2.5.4, https://github.com/MrOlm/drep) was used with the option ‘dereplicate_wf -p 16-comp 70-con 10-str100-strW 0’. MAGs were assessed by completeness and contamination using CheckM (v1.1.3, https://github.com/Ecogenomics/CheckM/wiki) with lineage-specific marker genes [20]. MAGs with completeness > 70% and contamination < 10% were used for downstream analysis. The abundances of MAGs were calculated by the average coverage depth over the assembled genome and were normalized to the number of million read pairs in each sample. The ANI was calculated using FastANI (v1.32). The program Circos was used to plot gene locations, GC skew and GC content (http://www.circos.ca) [61].

### Taxonomic assignment and phylogenetic analysis of MAGs

Taxonomic assignment was performed using AMPHORA2 (https://github.com/martinwu/AMPHORA2) [62]. Briefly, MAGs were phylogenetically assigned to taxonomies based on a set of 31 protein-encoding phylogenetic marker genes (*dnaG*, *frr*, *infC*, *nusA*, *pgk*, *pyrG*, *rplA*, *rplB*, *rplC*, *rplD*, *rplE*, *rplF*, *rplK*, *rplL*, *rplM*, *rplN*, *rplP*, *rplS*, *rplT*, *rpmA*, *rpoB*, *rpsB, rpsC*, *rpsE*, *rpsI*, *rpsJ*, *rpsK*, *rpsM*, *rpsS*, *smpB*, and *tsf*) from representatives of complete bacterial genomes [63]. The prokaryotic RefSeq genome dataset was downloaded from the NCBI RefSeq collections (release 99). Genomes composed of < 200 contigs with N50 > 20 kb and completeness and contamination > 95% and < 5%, respectively, were downloaded. Homologs of each of 31 marker genes were identified from these complete prokaryotic genomes and MAGs by HMMER3 with a cutoff of e value < 1 × 10^−10^ (v3.3 http://www.hmmer.org). The corresponding protein sequences were retrieved, aligned, trimmed, and concatenated by species in MEGA alignment (V7, https://www.megasoftware.net/). A phylogenetic tree was generated using the maximum parsimony method of RAxML (v8.2.4, https://cme.h-its.org/exelixis/software.html) [64] and visualized using the iTOL webtool (v6.1.2, https://itol.embl.de/) [65]. MEGA (v10.1.8) [66] was used to construct the topological tree using the maximum likelihood program.

### CAZyme annotation, polysaccharide utilization locus prediction, and metabolic analysis of MAGs

CAZy assignments were generated by comparing queries to the full-length sequences in the CAZy database using hmmscan (v2.41.1, https://www.ebi.ac.uk/Tools/hmmer/search/hmmscan/) with a cutoff of e value < 1 × 10^-5^. PULs were predicted following the protocol of PULDB [26] and were drawn using GenomeDiagram [67]. MAGs were annotated using eggNOG-mapper-1.03 in the EggNOG database (with *e* value < 1 × 10^−10^). After assignment of key genes, MAGs were assessed for specific pathways and functions based on the canonical pathways available in the KEGG Pathway Database (www.kegg.jp). The distribution of CAZymes and KEGG pathways across MAGs was visualized using the pheatmap package in R (https://www.r-project.org).

### Rumen tissue collection, dissociation, and single-cell RNA sequencing (scRNA-seq)

For single-cell RNA sequencing studies, the use of 1 to 3 samples is well accepted and widely adopted to reveal the cell type composition within one certain tissue [68, 69]. This is because cell type compositions of the same tissue type under the same biological conditions are similar and relatively stable [70]. Therefore, results from the small number of samples can be extrapolated to the larger cohort, which has been applied in mouse and human cell atlas research [70, 71]; the authors reported that the multi-donor analysis of one tissue had limited effects on cell-type discovery [70]. Thus, we selected three Holstein dairy cows used in our previous study [72] for scRNA-seq analysis. Briefly, tissues were collected from the ventral sac region of the rumen, the digesta were washed out using Dulbecco’s phosphate-buffered saline (DPBS), and rumen tissue samples were transferred to the laboratory with tissue storage solution (Miltenyi Biotec, Bergisch Gladbach, Germany) within 1 h. To prepare single-cell suspensions, rumen tissues were firstly minced into 10 × 0.5 mm^2^ pieces and incubated with 20 mM ethylene diamine tetra-acetic acid (EDTA) for 30 min. After rinsing with DPBS and chopping into 1-mm pieces, tissue pieces were transferred to a 15-mL centrifuge tube and incubated with 0.25% trypsin-EDTA (Gibco) in a 37 °C water bath for 5 min. Next, the centrifuge tube containing tissues was inserted into ice for 2 min, and prechilled HBSS was added to stop the digestion. The supernatant was discarded after centrifugation at 300×*g* for 2 min at 4 °C. After washing twice with cold HBSS, samples were treated with multiple enzymes (1.5 mg/ml of collagenase I, collagenase IV, and dispase along with 100 U/ml hyaluronidase and 50 U/ml DNase I) for 30 min at 37 °C. The digestion was stopped by adding 10% fatal bovine serum (FBS), and then followed by a filtration step through 70-μm and 30-μm SmartStrainer (Miltenyi Biotec, Bergisch Gladbach, Germany). Dissociated cells were centrifuged at 300×*g* for 5 min at 4 °C and resuspended in 2 mL of HBSS. After washing twice with 1 × PBS containing 0.04% BSA, the samples were removed dead cells and cellular debris using the MACS Dead Cell Removal Kit (Miltenyi Biotec, Bergisch Gladbach, Germany) following the manufacturer’s recommendations. Finally, single cell suspensions were diluted to 700–1200 cells/μL with 1 × PBS containing 0.04% BSA and loaded onto a 10× chip B and processed with the 10× Chromium controller. The library construction was performed using the Chromium Single Cell 3' Reagent Kits v3 (10× Genomics) according to the manufacturer’s instructions. Qualities of libraries were checked using the Agilent Bioanalyzer High Sensitivity chip (Agilent, Palo Alto, USA). Generated libraries were sequenced on a NovaSeq 6000 sequencing system (150 bp paired-ends).

### ScRNA-seq data processing, cell clustering, and differential gene analysis

Our previous study performed a preliminary analysis of all cells in the rumen, but many epithelial cell types were not annotated with exact identity [72]. Therefore, in this study, based on our previous raw single‐cell data, we deeply carried out the epithelial cell reclustering analysis and other secondary analysis. ScRNA-seq results were converted into fastq files using Illumina bcl2fastq software. cDNA reads were aligned to the ARS-USD1.2 (Bos_taurus ensemblV99) reference genome, and the gene expression matrix was built using Cell Ranger (v3.1.0). The Seurat package (v3.23) was used for cell filtering, data normalization, dimensionality reduction, clustering, and gene differential expression analysis. Cells with fewer than 500 genes or more than 4000 genes, UMI counts greater than 50,000, and a mitochondrial gene ratio over 40% were excluded. The DoubletFinder package (v2.0.3) was used to identify doublets. The “Normalization” and “FindVariableGenes” functions of the R package Seurat were used to calculate the expression values of genes and to identify variable genes, respectively. Subsequently, principal component analysis was performed, and Harmony analysis [73] was used for batch effect correction. Cell clustering was performed using the “FindClusters” function and visualized by uniform manifold approximation and projection (UMAP). A rumen epithelial single-cell map was generated by removing other cell types in the rumen with well-defined marker genes (Supplementary Figure S3) and reclustering epithelial cells with a resolution of 1.6. Next, the Wilcoxon test implemented in the “FindAllMarkers” function was performed to determine differentially expressed genes (DEGs) or markers (|‘avg_logFC’| > 0.25 and ‘*P*_adj’ < 0.05) of each cell cluster. The DEGs are presented in Supplementary Table S5.

To further distinguish cell subtypes of MC, BC, SC, and GC differed from each other respectively, we firstly subset the MC, BC, SC, and GC separately. Next, we used the “FindAllMarkers” function implemented in Seurat R package to determine differentially expressed genes (|‘avg_logFC’| > 0.25 and ‘*P*_adj’ < 0.05) of each cell subtype in MC, BC, SC, and GC separately. The representative highly expressed genes of each cell subtype were show in the Figures S5A–D.

### Gene set scoring analysis

“Short-chain fatty acid (SCFA/VFA) catabolic process” and “ketone body biosynthetic process” gene sets were obtained from the MSigDB database, and the genes within these gene sets are listed in Supplementary Table S9. The signature score of each gene set in each cell type was computed using the AddModuleScore function in the Seurat R package. The differences in the signature scores across cell types were evaluated by a two-sided Wilcoxon rank sum test. Mean values labeled without a common letter were defined as significantly different (the order of the letters (from “a” to “l”) was sorted according to mean value from high to low, adjusted *P* value < 0.05).

### Immunofluorescence

Immunofluorescence was performed on 5-μm-thick, formalin-fixed, paraffin-embedded rumen tissue slides. In brief, antigen retrieval was performed by microwaving the slides at 98 °C in 10 mM Tris-EDTA (pH 8.0). The 3% methanol-hydrogen peroxide solution was used to block the slides at room temperature for 25 min. Next, slides were washed three times (5 min each time) using PBS (pH 7.4). The primary antibody was added to the slides for incubation at 4 °C overnight after blocking with 0.5% BSA for 30 min. Slides were incubated with polymer horseradish peroxidase (HRP)-conjugated antibody specific to rabbits after washing with PBS (pH 7.4). After rinsing, CY3-TSA or FITC-TSA was added to each slide for incubation at room temperature. Antigen retrieval was accomplished on stained slides to prepare them for staining to detect the next target protein. Slides were counterstained with DAPI. The primary antibodies used were anti-GJA1 (ER1802-88, HUABIO) and anti-KRT6A (ET1611-70, HUABIO). Mages were acquired using the Olympus BX63 microscope.

### Inferring microbiome-host interactions

We used MicrobioLink, a computational pipeline, to predict the cross-talks between the 30 key MAGs and the host cells based on the domain–domain-based protein–protein interaction. Prodigal (v2.6.3, https://github.com/hyattpd/Prodigal) was used for amino acid sequences prediction based on all the 30 key MAGs. Protein domain annotation against Pfam database (v33.1, https://anaconda.org/bioconda/pfam_scan) was performed using Pfam_scan (v1.6, https://anaconda.org/bioconda/pfam_scan), and secreting proteins were further selected and used for downstream analysis (Supplementary Table S6). The bovine proteins located in the cellular membrane and extracellular matrix were identified using UniProt [49]. The bovine proteins domain annotation was obtained using the “hmmscan” function implemented in the HMMER software (v3.3) against Pfam database (Supplementary Table S7). Then, we used the domain-domain interaction prediction (DDI) function of the MicrobioLink to predict the microbiome-host interactions based on domains contained in the proteins. The signaling networks from host receptors to the target genes were compiled using the network diffusion model inferred by TieDie implemented in MicrobioLink.

## Supplementary Information

**Additional file 1: Supplementary Table S1.** Summary of sequence data generated from rumen samples of 49 dairy cows. **Supplementary Table S2.** Summary of rumen bacterial genomes recovered from 49 dairy cows. **Supplementary Table S3.** Numbers of each CAZyme family predicted in the total metagenome. **Supplementary Table S4.** PUL profile of MAGs in *Bacteroidetes*. **Supplementary Table S5.** DEGs of each rumen epithelial cell type. **Supplementary Table S6.** List of microbial proteins derived from the 30 key MAG sequence data. **Supplementary Table S7.** Localization-filtered bovine receptor proteins. **Supplementary Table S8.** Interactions identified using domain–domain-based protein–protein predictions between microbial and bovine receptor proteins. **Supplementary Table S9.** Genes of short-chain fatty acid catabolic process and ketone body biosynthetic process gene sets.

**Additional file 2: Supplementary Figure S1.** Taxonomic composition of bacterial genomes at different taxonomic levels. **Supplementary Figure S2.** Examples of selected PULs predicted in MAGs belonging to *Bacteroidetes*. A: the most common PULs, a susC/susD pair. B, C, D and E: PULs with similar configurations involved in xylan degradation. F and G: PULs involved in pectin degradation. **Supplementary Figure S3.** Epithelial cell clusters were selected for repeat cluster analysis (second-level analysis) based on the markers *KRT14*, *KRT5*, and *KRT19* in the rumen single-cell landscape. **Supplementary Figure S4.** The similarity between the single-cell data of the 3 cows’ rumen epithelial cells. (A) The multi-donor analysis showed cells from the 3 cows were well overlapped. (B) The analysis of similarities (ANOSIM) on the single-cell data after batch effect removal of the 3 cows through a distance measure using the vegan R package (v2.5-7) showed the ANOSIM statistic R was -0.012 and the P value was 1 indicating cells from the 3 cows overlapped well. **Supplementary Figure S5.** The highly expressed genes in each cell subtype of rumen epithelial cells. (A-D) Heatmap showing the representative highly expressed genes of cell subtypes of MC (A), BC (B), GC (C), and SC (D). (E) The UMAP maps of rumen epithelial single-cell data with cells colored by expression of genes *GSTA1*, *TMEM79*, and *FABP4* for channel-gap like spinous cells (cg-like SCs). Gene expression levels are indicated in shades of red. MC: Mitotic cell; BC: Basal cell; GC: Granule cell; SC: Spinous cell.

## Data Availability

The metagenome sequences of 49 rumen samples were deposited into the NCBI Sequence Read Archive (SRA) under the accession numbers PRJNA526070 and PRJNA597489. The fasta files of all 186 MAGs were deposited into the NCBI SRA under the accession number PRJNA730102. The raw single-cell data were collected from our previous study [72]  and the processed sequencing data from this study were deposited in the Gene Expression Omnibus (GEO) database (accession number GSE175652), and other relevant data are available upon request.

## Abbreviations

BC: Basal cell
CAZyme: Carbohydrate active enzymes
CBM: Carbohydrate-binding modules
CE: Carbohydrates esterases
GC: Granule cell
GH: Glycoside hydrolases
GT: Glycosyl transferases
MAGs: Metagenome-assembled genomes
MC: Mitotic cell
PUL: Polysaccharide utilization loci
SC: Spinous cell
scRNA-seq: Single-cell RNA sequencing
VFAs: Volatile fatty acids

## References

[1] Kearney J (2010). Food consumption trends and drivers. Phil Trans Roy Soc London B..

[2] FAO. Food Outlook: biannual report on global food markets Food and Agriculture Organization of the United Nations. Accessed May. 2019.

[3] Hess M, Sczyrba A, Egan R, Kim T-W, Chokhawala H, Schroth G (2011). Metagenomic discovery of biomass-degrading genes and genomes from cow rumen. Science..

[4] Deng W, Xi D, Mao H, Wanapat M (2008). The use of molecular techniques based on ribosomal RNA and DNA for rumen microbial ecosystem studies: a review. Mol Biol Rep..

[5] Shabat SKB, Sasson G, Doronfaigenboim A, Durman T, Yaacoby S, Miller MEB, et al. Specific microbiome-dependent mechanisms underlie the energy harvest efficiency of ruminants. ISME J. 2016. 10.1038/ismej.2016.62 PMC5148187 27152936

[6] Sun HZ, Zhou M, Wang O, Chen Y, Liu JX, Guan LL (2020). Multi-omics reveals functional genomic and metabolic mechanisms of milk production and quality in dairy cows. Bioinformatics..

[7] Li F, Guan LL (2017). Metatranscriptomic profiling reveals linkages between the active rumen microbiome and feed efficiency in beef cattle. Appl Environ Microbiol..

[8] Li F, Hitch TCA, Chen Y, Creevey CJ, Guan LL (2019). Comparative metagenomic and metatranscriptomic analyses reveal the breed effect on the rumen microbiome and its associations with feed efficiency in beef cattle. Microbiome..

[9] Xue M-Y, Sun H-Z, Wu X-H, Liu J-X, Guan LL (2020). Multi-omics reveals that the rumen microbiome and its metabolome together with the host metabolome contribute to individualized dairy cow performance. Microbiome..

[10] Stewart RD, Auffret MD, Warr A, Walker AW, Roehe R, Watson M (2019). Compendium of 4,941 rumen metagenome-assembled genomes for rumen microbiome biology and enzyme discovery. Nat Biotechnol..

[11] Seshadri R, Leahy SC, Attwood GT, Teh KH, Lambie SC, Cookson AL (2018). Cultivation and sequencing of rumen microbiome members from the Hungate1000 Collection. Nat Biotechnol..

[12] Stewart RD, Auffret MD, Warr A, Wiser AH, Press MO, Langford KW (2018). Assembly of 913 microbial genomes from metagenomic sequencing of the cow rumen. Nat Commun..

[13] Steele MA, Croom J, Kahler M, AlZahal O, Hook SE, Plaizier K (2011). Bovine rumen epithelium undergoes rapid structural adaptations during grain-induced subacute ruminal acidosis. Ame J Physiol Regul Integr Comp Physiol..

[14] Yohe T, Tucker H, Parsons C, Geiger A, Akers R, Daniels K (2016). Initial evidence supporting existence of potential rumen epidermal stem and progenitor cells. J Dairy Sci..

[15] Kent-Dennis C, Penner G (2021). Effects of a proinflammatory response on metabolic function of cultured, primary ruminal epithelial cells. J Dairy Sci..

[16] Stumpff F (2018). A look at the smelly side of physiology: transport of short chain fatty acids. Pflügers Arch-Eur J Phy..

[17] Xiang R, Oddy VH, Archibald AL, Vercoe PE, Dalrymple BP (2016). Epithelial, metabolic and innate immunity transcriptomic signatures differentiating the rumen from other sheep and mammalian gastrointestinal tract tissues. PeerJ..

[18] Macosko EZ, Basu A, Satija R, Nemesh J, Shekhar K, Goldman M (2015). Highly parallel genome-wide expression profiling of individual cells using nanoliter droplets. Cell..

[19] Tang F, Barbacioru C, Wang Y, Nordman E, Lee C, Xu N (2009). mRNA-Seq whole-transcriptome analysis of a single cell. Nat methods..

[20] Parks DH, Imelfort M, Skennerton CT, Hugenholtz P, Tyson GW (2015). CheckM: assessing the quality of microbial genomes recovered from isolates, single cells, and metagenomes. Genome Res..

[21] Creevey CJ, Kelly WJ, Henderson G, Leahy SC (2014). Determining the culturability of the rumen bacterial microbiome. Microb Biotechnol..

[22] Kim M, Morrison M, Yu Z (2011). Status of the phylogenetic diversity census of ruminal microbiomes. Fems Microbiol Ecol..

[23] Bjursell MK, Martens EC, Gordon JI (2006). Functional genomic and metabolic studies of the adaptations of a prominent adult human gut symbiont, Bacteroides thetaiotaomicron, to the suckling period. J Biol Chem..

[24] Solden LM, Naas AE, Roux S, Daly RA, Collins WB, Nicora CD (2018). Interspecies cross-feeding orchestrates carbon degradation in the rumen ecosystem. Nat Microbiol..

[25] Martens EC, Koropatkin NM, Smith TJ, Gordon JI (2009). Complex glycan catabolism by the human gut microbiota: the Bacteroidetes Sus-like paradigm. J Biol Chem..

[26] Terrapon N, Lombard V, Gilbert HJ, Henrissat B (2015). Automatic prediction of polysaccharide utilization loci in Bacteroidetes species. Bioinformatics..

[27] Bryant MP, Small N, Bouma C, Chu H (1958). Bacteroides ruminicola n. sp. and Succinimonas amylolytica; the new genus and species; species of succinic acid-producing anaerobic bacteria of the bovine rumen. J Bacteriol..

[28] Avgustin G, Wallace RJ, Flint HJ (1997). Phenotypic diversity among ruminal isolates of Prevotella ruminicola: proposal of Prevotella brevis sp. nov., Prevotella bryantii sp. nov., and Prevotella albensis sp. nov. and redefinition of Prevotella ruminicola. Int J Syst Bacteriol..

[29] Roehe R, Dewhurst RJ, Duthie CA, Rooke JA, Mckain N, Ross DW (2016). Bovine host genetic variation influences rumen microbial methane production with best selection criterion for low methane emitting and efficiently feed converting hosts based on metagenomic gene abundance. PLoS Genet..

[30] Hernandez-Sanabria E, Goonewardene LA, Wang Z, Durunna ON, Moore SS (2012). Guan, et al. Impact of feed efficiency and diet on adaptive variations in the bacterial community in the rumen fluid of cattle. Appl Environ Microbiol..

[31] Rooke JA, Wallace RJ, Duthie C-A, McKain N, de Souza SM, Hyslop JJ (2014). Hydrogen and methane emissions from beef cattle and their rumen microbial community vary with diet, time after feeding and genotype. Brit J Nutr..

[32] Zou Z, Long X, Zhao Q, Zheng Y, Song M (2020). Ma S, et al.

[33] He H, Suryawanshi H, Morozov P, Gay-Mimbrera J, Del Duca E, Kim HJ (2020). Single-cell transcriptome analysis of human skin identifies novel fibroblast subpopulation and enrichment of immune subsets in atopic dermatitis. J Allergy Clin Immunol..

[34] Robitaille H, Proulx R, Robitaille K, Blouin R, Germain L (2005). The mitogen-activated protein kinase kinase kinase dual leucine zipper-bearing kinase (DLK) acts as a key regulator of keratinocyte terminal differentiation. J Biol Chem..

[35] CHANAS SA, JIANG Q, McMAHON M, McWALTER GK, McLELLAN LI, ELCOMBE CR (2002). Loss of the Nrf2 transcription factor causes a marked reduction in constitutive and inducible expression of the glutathione S-transferase Gsta1, Gsta2, Gstm1, Gstm2, Gstm3 and Gstm4 genes in the livers of male and female mice. Biochem J..

[36] Saunders SP, Floudas A, Moran T, Byrne CM, Rooney MD, Fahy CM (2020). Dysregulated skin barrier function in Tmem79 mutant mice promotes IL-17A-dependent spontaneous skin and lung inflammation. Allergy..

[37] Shi H, Zhang C, Zhao W, Luo J, Loor J (2017). Peroxisome proliferator-activated receptor delta facilitates lipid secretion and catabolism of fatty acids in dairy goat mammary epithelial cells. J Ddairy Sci..

[38] Aschenbach JR (2009). Bilk S, Tadesse G, Stumpff F, Gäbel G. Bicarbonate-dependent and bicarbonate-independent mechanisms contribute to nondiffusive uptake of acetate in the ruminal epithelium of sheep. Am J Physiol Gastrointest Liver Physiol..

[39] Zhao K, Chen Y, Penner G, Oba M, Guan L (2017). Transcriptome analysis of ruminal epithelia revealed potential regulatory mechanisms involved in host adaptation to gradual high fermentable dietary transition in beef cattle. BMC Genomics..

[40] Malmuthuge N, Liang G (2019). Regulation of rumen development in neonatal ruminants through microbial metagenomes and host transcriptomes. Genome Biol..

[41] Muller F, Huber K, Pfannkuche H, Aschenbach JR, Breves G, Gabel G (2002). Transport of ketone bodies and lactate in the sheep ruminal epithelium by monocarboxylate transporter 1. Am J Physiol Gastrointest Liver Physiol..

[42] Pan X, Cai Y, Li Z, Chen X, Heller R, Wang N, et al. Modes of genetic adaptations underlying functional innovations in the rumen. Sci China Life Sci. 2020:1–21. 10.1007/s11427-020-1828-8 33165812

[43] Baaske L, Gäbel G, Dengler F (2020). Ruminal epithelium: A checkpoint for cattle health. J Dairy Res..

[44] Pizzagalli MD, Bensimon A, Superti-Furga G (2021). A guide to plasma membrane solute carrier proteins. FEBS J..

[45] Gäbel G, Aschenbach J, Müller F (2002). Transfer of energy substrates across the ruminal epithelium: implications and limitations. Anim Health Res Rev..

[46] Sudhakar P, Jacomin AC, Hautefort I, Samavedam S, Fatemian K, Ari E (2019). Targeted interplay between bacterial pathogens and host autophagy. Autophagy..

[47] Wojcik J, Schächter V (2001). Protein-protein interaction map inference using interacting domain profile pairs. Bioinformatics..

[48] Andrighetti T, Bohar B, Lemke N, Sudhakar P, Korcsmaros T. MicrobioLink: An Integrated Computational Pipeline to Infer Functional Effects of Microbiome-Host Interactions. Cells. 2020;9. 10.3390/cells9051278 PMC7291277 32455748

[49] Consortium TU (2020). UniProt: the universal protein knowledgebase in 2021. Nucleic Acids Res..

[50] Shen JS, Chai Z, Song LJ, Liu JX, Wu YM (2012). Insertion depth of oral stomach tubes may affect the fermentation parameters of ruminal fluid collected in dairy cows. J Dairy Sci..

[51] Xue MY, Sun HZ, Wu XH, Guan LL, Liu JX (2018). Assessment of rumen microbiota from a large dairy cattle cohort reveals the pan and core bacteriomes contributing to varied phenotypes. Appl Environ Microbiol..

[52] Xue MY, Sun HZ, Wu XH, Guan LL, Liu JX (2019). Assessment of rumen bacteria in dairy cows with varied milk protein yield. J Dairy Sci..

[53] Yu Z, Morrison M (2004). Improved extraction of PCR-quality community DNA from digesta and fecal samples. BioTechniques..

[54] Li D, Liu C-M, Luo R, Sadakane K, Lam T-W (2015). MEGAHIT: an ultra-fast single-node solution for large and complex metagenomics assembly via succinct de Bruijn graph. Bioinformatics..

[55] Noguchi H, Park J, Takagi T (2006). MetaGene: prokaryotic gene finding from environmental genome shotgun sequences. Nucleic Acids Res..

[56] Niu B, Fu L, Wu S, Li W, Zhu Z (2012). CD-HIT: accelerated for clustering the next-generation sequencing data. Bioinformatics..

[57] Yu C, Wang J, Kristiansen K, Li R, Yiu S-M, Lam T-W (2009). SOAP2: an improved ultrafast tool for short read alignment. Bioinformatics..

[58] Edgar RC (2010). Search and clustering orders of magnitude faster than BLAST. Bioinformatics..

[59] Buchfink B, Xie C, Huson DH (2014). Fast and sensitive protein alignment using DIAMOND. Nat Methods..

[60] Kang DD, Froula J, Egan R, Wang Z (2015). MetaBAT, an efficient tool for accurately reconstructing single genomes from complex microbial communities. PeerJ..

[61] Krzywinski M, Schein J, Birol I, Connors J, Gascoyne R, Horsman D (2009). Circos: an information aesthetic for comparative genomics. Genome Res..

[62] Wu M, Scott AJ (2012). Phylogenomic analysis of bacterial and archaeal sequences with AMPHORA2. Bioinformatics..

[63] Wu M, Eisen JA. A simple, fast, and accurate method of phylogenomic inference. Genome Biol. 2008;9:R151–1. 10.1186/gb-2008-9-10-r151 PMC2760878 18851752

[64] Stamatakis A (2006). RAxML-VI-HPC: maximum likelihood-based phylogenetic analyses with thousands of taxa and mixed models. Bioinformatics..

[65] Letunic I, Bork P (2016). Interactive tree of life (iTOL) v3: an online tool for the display and annotation of phylogenetic and other trees. Nucleic Acids Res..

[66] Kumar S, Stecher G, Li M, Knyaz C, Tamura K (2018). MEGA X: molecular evolutionary genetics analysis across computing platforms. Mol Biol Evol..

[67] Pritchard L, White JA, Birch PR, Toth IK (2006). GenomeDiagram: a python package for the visualization of large-scale genomic data. Bioinformatics..

[68] Guo J, Grow EJ, Mlcochova H, Maher GJ, Lindskog C, Nie X (2018). The adult human testis transcriptional cell atlas. Cell Res..

[69] Wang Y, Song W, Wang J, Wang T, Xiong X, Qi Z, et al. Single-cell transcriptome analysis reveals differential nutrient absorption functions in human intestine. J Exp Med. 2020;217. 10.1084/jem.20191130 PMC7041720 31753849

[70] Han X, Zhou Z, Fei L, Sun H, Wang R, Chen Y (2020). Construction of a human cell landscape at single-cell level. Nature..

[71] Han X, Wang R, Zhou Y, Fei L, Sun H, Lai S (2018). Mapping the mouse cell atlas by Microwell-Seq. Cell..

[72] Wu JJ, Zhu SL, Gu FF, Valencak TG, Liu JX, Sun HZ. Cross-tissue single-cell transcriptomic landscape reveals the key cell subtypes and their potential roles in the nutrient absorption and metabolism in dairy cattle. J Adv Res. 2021. 10.1016/j.jare.2021.11.009. 10.1016/j.jare.2021.11.009 PMC9039752 35499046

[73] Korsunsky I, Millard N, Fan J, Slowikowski K, Zhang F, Wei K (2019). Fast, sensitive and accurate integration of single-cell data with Harmony. Nat Methods..

