# Supplementary material for: Investigation of fiber utilization in the rumen of dairy cows based on metagenome-assembled genomes and single-cell RNA sequencing
Source: Microbiome. 2022 Jan 20;10:11. doi: 10.1186/s40168-021-01211-w (PMC8772221; doi:10.1186/s40168-021-01211-w)
Supplement: Supplementary file 3 — Additional file 2: Supplementary Figure S1. Taxonomic composition of bacterial genomes at different taxonomic levels. Supplementary Figure S2. Examples of selected PULs predicted in MAGs belonging to Bacteroidetes. A: the most common PULs, a susC/susD pair. B, C, D and E: PULs with similar configurations involved in xylan degradation. F and G: PULs involved in pectin degradation. Supplementary Figure S3. Epithelial cell clusters were selected for repeat cluster analysis (second-level analysis) based on the markers KRT14, KRT5, and KRT19 in the rumen single-cell landscape. Supplementary Figure S4. The similarity between the single-cell data of the 3 cows’ rumen epithelial cells. (A) The multi-donor analysis showed cells from the 3 cows were well overlapped. (B) The analysis of similarities (ANOSIM) on the single-cell data after batch effect removal of the 3 cows through a distance measure using the vegan R package (v2.5-7) showed the ANOSIM statistic R was -0.012 and the P value was 1 indicating cells from the 3 cows overlapped well. Supplementary Figure S5. The highly expressed genes in each cell subtype of rumen epithelial cells. (A-D) Heatmap showing the representative highly expressed genes of cell subtypes of MC (A), BC (B), GC (C), and SC (D). (E) The UMAP maps of rumen epithelial single-cell data with cells colored by expression of genes GSTA1, TMEM79, and FABP4 for channel-gap like spinous cells (cg-like SCs). Gene expression levels are indicated in shades of red. MC: Mitotic cell; BC: Basal cell; GC: Granule cell; SC: Spinous cell. [file 40168_2021_1211_MOESM3_ESM.docx]

**Supplementary Figure S1. Taxonomic composition of bacterial genomes at different taxonomic levels.**

**Supplementary Figure S2.** **Examples of the selected PULs predicted in MAGs belonged to *Bacteroidetes*.** A: the most common PULs, a susC/susD pair. B, C, D and E: PULs with similar configurations involved in xylan degradation. F and G: PULs involved in pectin degradation.


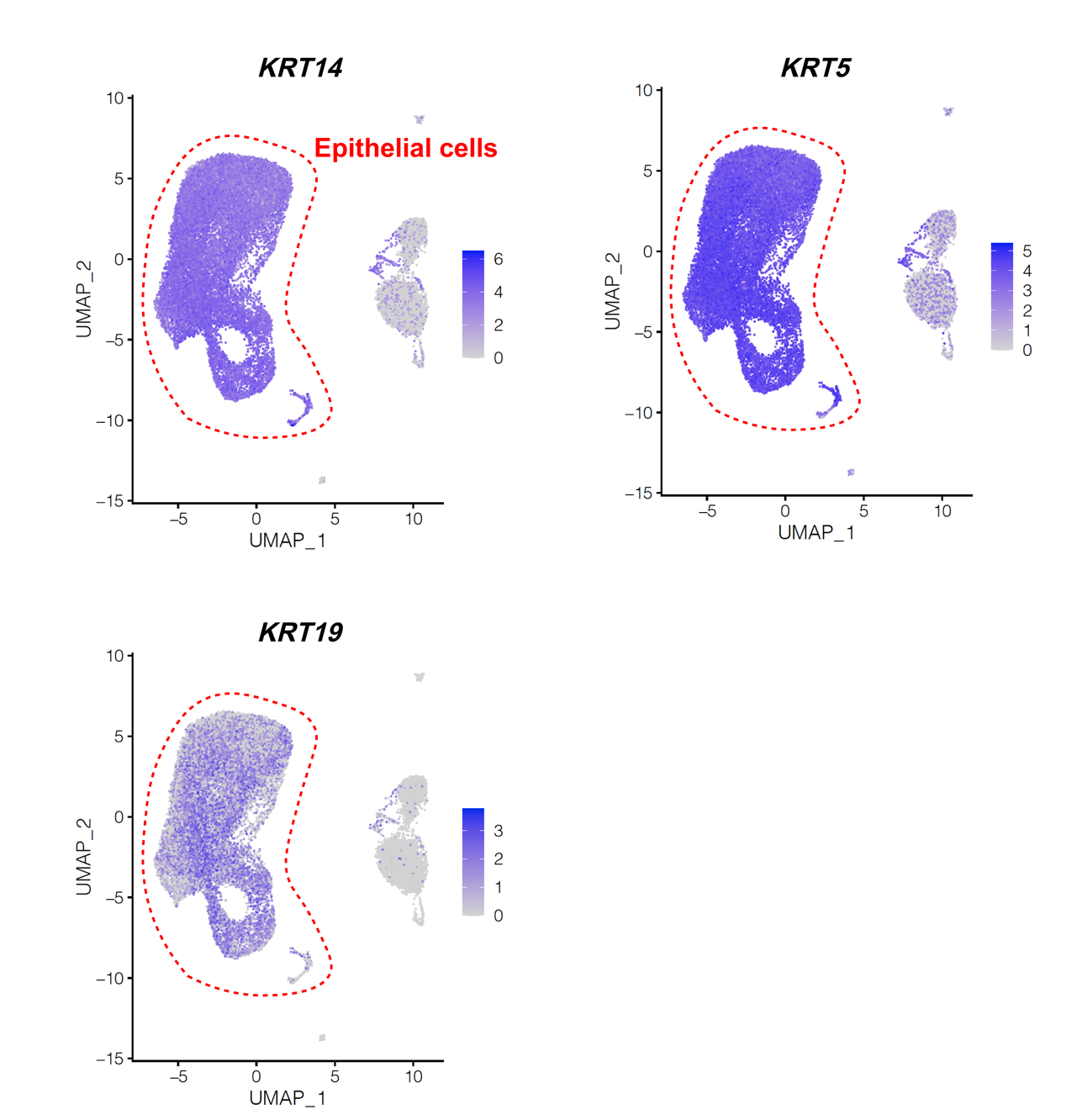


**Supplementary Figure S3. The epithelial cell clusters were selected to repeat cluster analysis (second-level analysis) based on the markers *KRT14*, *KRT5*, and *KRT19* in the rumen single-cell landscape.**


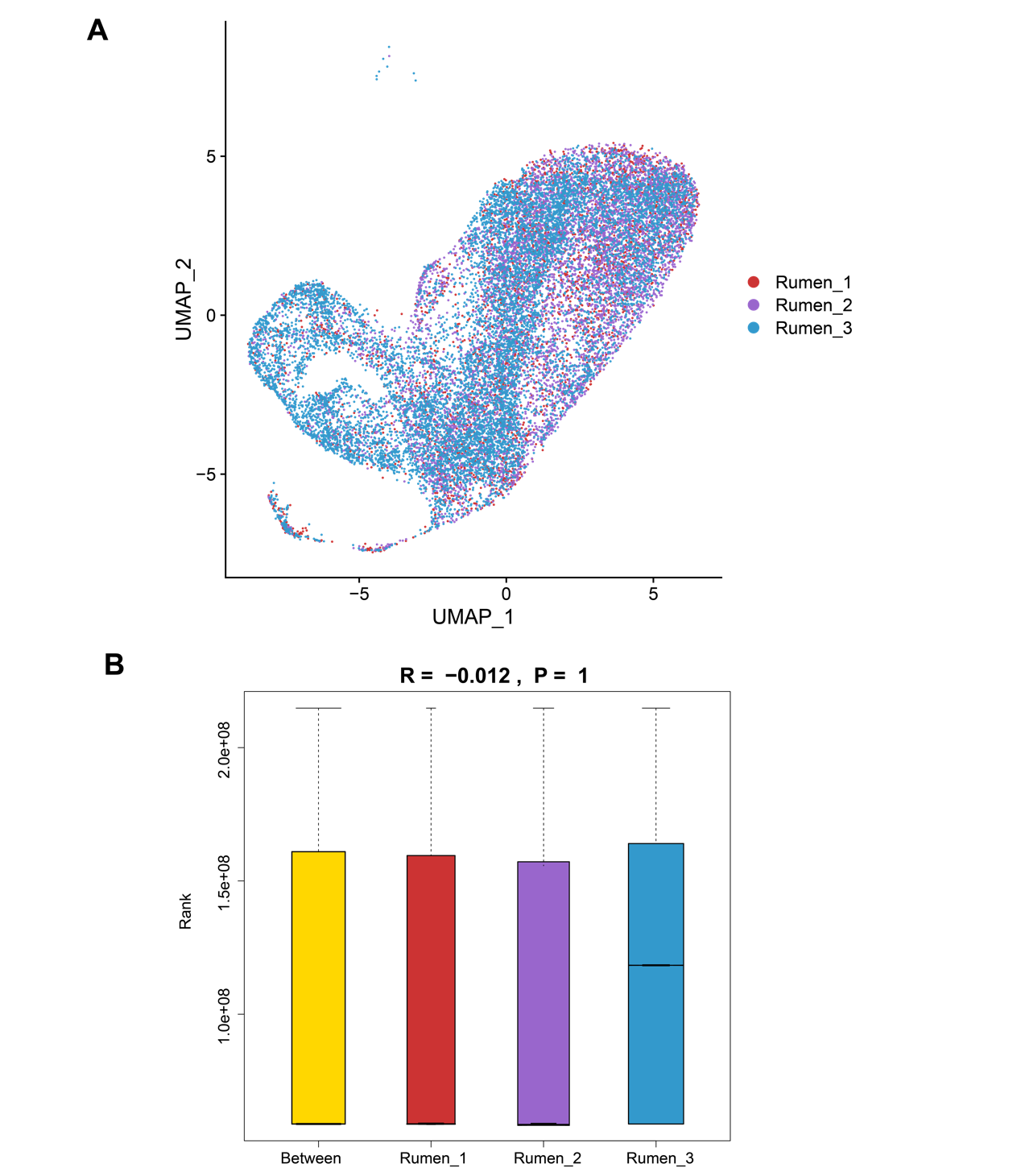


**Supplementary Figure S4. The similarity between the single-cell data of the 3 cows’ rumen epithelial cells. (A)** The multi-donor analysis showed cells from the 3 cows were well overlapped. **(B)** The analysis of similarities (ANOSIM) on the single-cell data after batch effect removal of the 3 cows through a distance measure using the vegan R package (v2.5-7) showed the ANOSIM statistic R was -0.012 and the *P* value was 1 indicating cells from the 3 cows overlapped well.


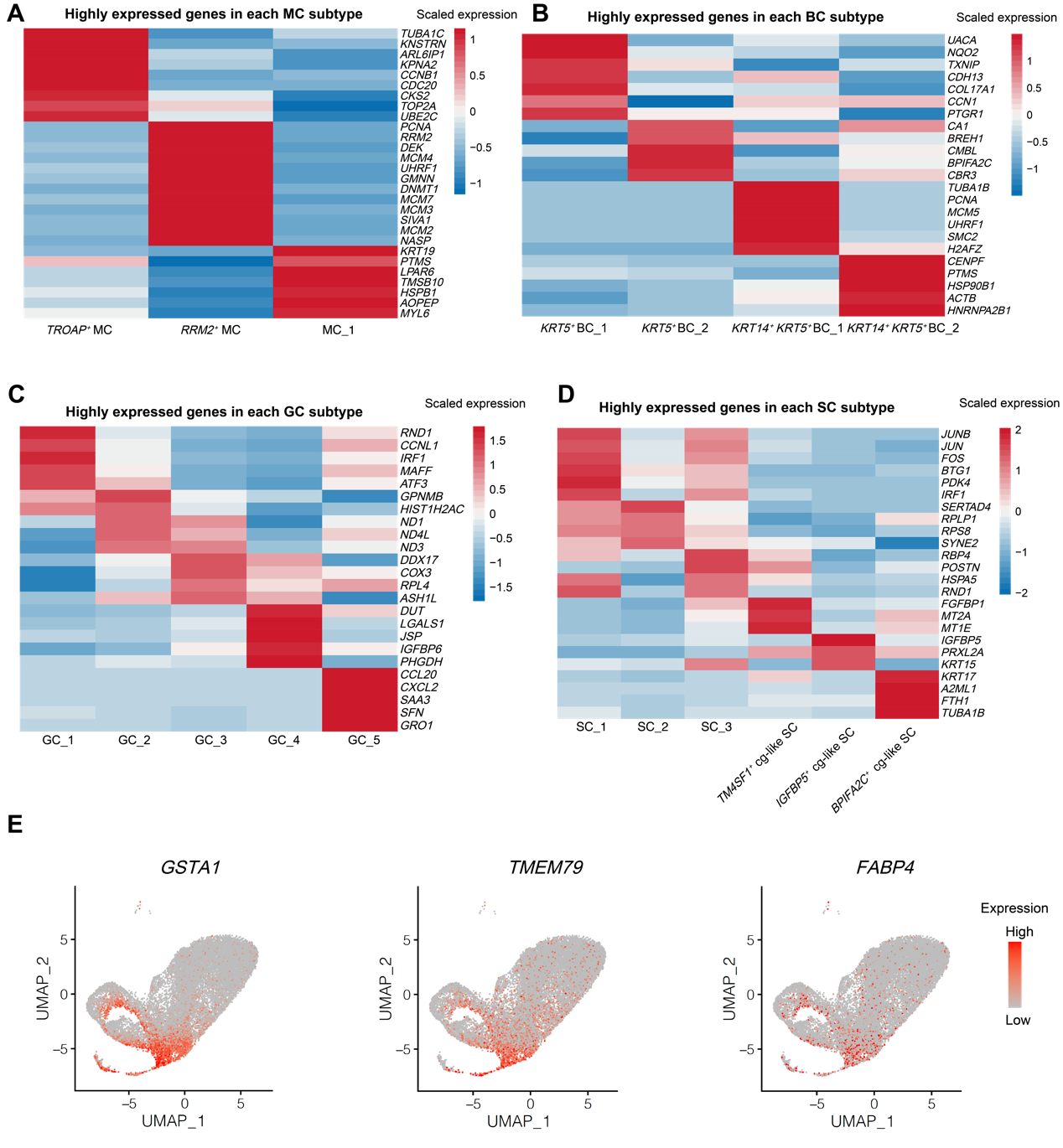


**Supplementary Figure S5. The highly expressed genes in each cell subtype of rumen epithelial cells. (A-D)** Heatmap showing the representative highly expressed genes of cell subtypes of MC (**A**), BC (**B**), GC (**C**), and SC (**D**). (**E**) The UMAP maps of rumen epithelial single-cell data with cells colored by expression of genes *GSTA1*, *TMEM79*, and *FABP4* for channel-gap like spinous cells (cg-like SCs). Gene expression levels are indicated in shades of red. MC: Mitotic cell; BC: Basal cell; GC: Granular cell; SC: Spinous cell.
